# The Relationship between Body Composition and a Gluten Free Diet in Children with Celiac Disease

**DOI:** 10.3390/nu10111817

**Published:** 2018-11-21

**Authors:** Paweł Więch, Zdzisława Chmiel, Dariusz Bazaliński, Izabela Sałacińska, Anna Bartosiewicz, Artur Mazur, Bartosz Korczowski, Monika Binkowska-Bury, Mariusz Dąbrowski

**Affiliations:** 1 Institute of Nursing and Health Sciences, Faculty of Medicine, University of Rzeszów, 35959 Rzeszów, Poland; zchmiel77@gmail.com (Z.C.); darek.bazalinski@wp.pl (D.B.); izabela.salacinska@wp.pl (I.S.); ania.bartosiewicz@gmail.com (A.B.); mbinkowskabury@gmail.com (M.B.-B.); mariusz.dabrowski58@gmail.com (M.D.); 2 Pediatric Department, Clinical Provincial Hospital No. 2 in Rzeszów, Faculty of Medicine, University of Rzeszów, 35301 Rzeszów, Poland; drmazur@poczta.onet.pl (A.M.); korczowski@op.pl (B.K.); 3 Diabetic Outpatient Clinic, Medical Center “Beta-Med” Rzeszów, 35073 Rzeszów, Poland

**Keywords:** celiac disease, body composition, gluten free diet, children

## Abstract

The primary and proven therapy, in cases of celiac disease (CD), is a rigorous gluten-free diet (GFD). However, there are reports of its negative effects in the form of nutritional deficiencies, obesity, and adverse changes in body composition. The study aimed to assess the impact of a GFD on the body composition of children with CD. In a case-controlled study (*n* = 41; mean age 10.81 y; SD = 3.96) children with CD, in various stages of treatment, underwent medical assessment. The control group consisted of healthy children and adolescents, strictly matched for gender and age in a 1:1 case-control manner. More than half of the examined children (*n* = 26) followed a GFD. CD children had significantly higher mean values of the fat free mass (FFM% = 80.68 vs. 76.66, *p* = 0.015), and total body water (TBW% = 65.22 vs. 60.47, *p* = 0.012), and lower mean values of the fat mass (FM% = 19.32 vs. 23.34, *p* = 0.015). Children who were on a GFD presented slightly higher, but not statistically significant, mean values of FM and FFM, than children who did not follow dietary recommendations (FM [kg] = 7.48 vs. 5.24, *p* = 0.064; FM% = 20.81 vs. 16.73, *p* = 0.087; FFM [kg] = 28.19 vs. 22.62, *p* = 0.110). After minimum one year of a GFD, CD children showed significantly higher values of FFM [kg] (*p* = 0.001), muscle mass (MM) [kg] (*p* < 0.001), TBW [L] (*p* < 0.001) and body cell mass (BCM) [kg] (*p* < 0.001). Furthermore, CD children who were on a GFD presented a significantly higher increase in weight (*p* = 0.034) and body mass index (BMI) (*p* = 0.021). The children adhering to a GFD demonstrate a tendency towards higher indices of selected body composition components.

## 1. Introduction

Celiac disease (CD) is a diet-dependent disease and one of the most common food intolerances in Europe posing significant health-related problems [1]. The disease may manifest itself at any age, yet it is frequently diagnosed in children up to 5 years of age, three in four cases being identified in female subjects. It is estimated that for each case diagnosed, there are 5 undetected cases [2], which is partly linked to the high prevalence of subclinical CD [3]. The expected global prevalence of CD is in the range from 0.2% to 5.6% [4]. In Europe, the relevant rate generally varies from 0.5% to 1% (in some countries reaching the level of 3%), and in Poland CD affects approximately 0.8% of the whole population. In the age group of 2.5–15 years, the condition affects 1 in 80 to 1 in 300 children [5,6].

CD is a life-long autoimmune enteropathy due to gluten sensitivity [7]. In CD patients the ingestion of gluten leads to an enteropathy with an impairment of the mucosal surface and abnormal absorption of nutrients [8]. In the case of patients with diagnosed CD, basic therapy involves the life-long adherence to a gluten free diet (GFD). The diet is designed to eliminate any type of food, drink or even medication containing wheat, rye or barley [9,10]. Early diagnosis and treatment make it possible to prevent numerous complications and to effectively eliminate physical and mental development impairments in children with this condition. Compliance with dietary recommendations closely correlates with symptom relief, improved condition of the mucous membrane, and consequently the patient’s improved nutritional status. Some studies confirm the effectiveness of a GFD in patients with celiac disease [9,11], yet other researchers argue that the use of this diet alone may contribute to nutritional deficiencies or to excessive body mass [12,13,14]. Therefore, systematic monitoring of both the nutritional status and body composition appears to be an important part of the therapy in CD [15,16]. The monitoring of dietary compliance is associated with a high chance of healing intestinal lesions and correction of specific body compositional abnormalities, expressed by normalizing fat mass, muscle mass, and bone mass. [16,17,18]. The changes in lean body mass take longer and, probably, are related more to improvement in the inflammatory state than to changes in absorption of food intake [16]. 

In practice, body composition assessment can be based on measurements of skin fold thickness, and methods of bioelectrical impedance (BIA) and dual-energy X-ray absorptiometry (DXA) [19,20,21]. Currently, there are no studies assessing body composition in children with CD. The scarcity of documentation describing body composition in patients with CD, representing varied age groups (at the time of the diagnosis, and during the nutritional therapy), provides for ambiguous evidence and leads to a difficulty in the ability to make comparisons [22,23]. The lack of unanimity of opinion in this regard suggests a need for further research and analyses, in particular, related to children and adolescents. Given the above, the present study was designed to assess the effects of a GFD in the body composition in children with CD.

## 2. Materials and Methods

### 2.1. Ethics

The study was approved by the institutional Bioethics Committee at the University of Rzeszów (Resolution No. 5/02/2012) and by all appropriate administrative bodies. The study was conducted in accordance with ethical standards laid down in an appropriate version of the Declaration of Helsinki and in Polish national regulations. The study was conducted according to the Strengthening the Reporting of Observational Studies in Epidemiology (STROBE) criteria.

### 2.2. Subjects

The study involved 41 children and adolescents (20 girls, 48.7%) with celiac disease receiving inpatient treatment at the Clinical Department of Pediatrics with the Pediatric Neurology Unit, at the Clinical Regional Hospital No. 2 in Rzeszów. The study group consisted of patients with newly diagnosed CD and subjects at different stages of treatment.

The inclusion criteria were as follows: diagnosed celiac disease, age 4 to 18 years, no other autoimmune or chronic diseases affecting height, weight or nutritional status, as well as written informed consent, signed by parents or legal guardians, and by the adolescents over 16 years of age.

**Figure 1:**
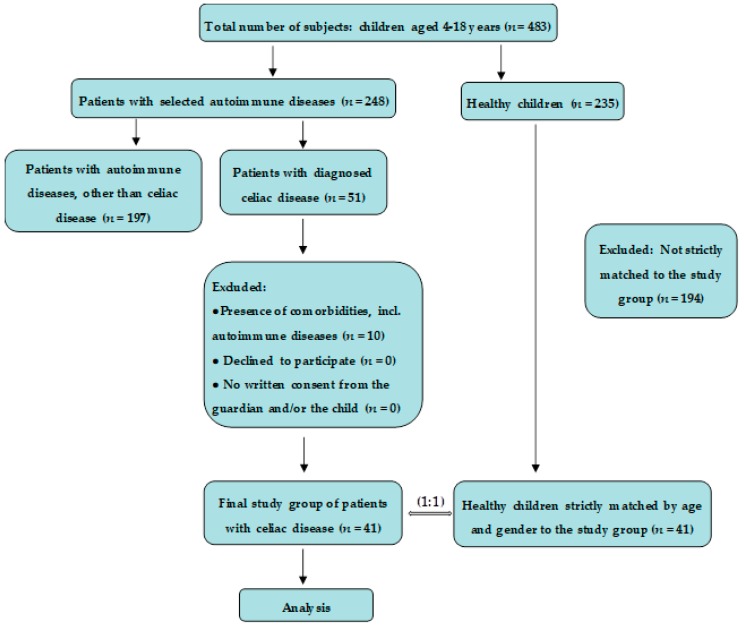
Flow chart demonstrating study participants selection.

The control group consisted of the same number of children and adolescents attending primary, middle, and secondary schools in urban and rural areas. Inclusion criteria were the same as for the study group with the exception for CD diagnosis. The healthy participants and those with CD were strictly matched for age (the nearest birth date) and gender in a 1:1 case-control manner. Figure 1 presents the recruitment process for the study group and the controls, while Table 1 shows the characteristics of the groups.

Detailed information concerning the children with CD, namely the diagnosis, the course and treatment of the disease, and comorbidities, was retrieved from their medical records. In addition, laboratory tests (level of IgA class anti-tissue transglutaminase antibodies TTG, level of IgA anti-endomysium antibodies EmA, and level of IgA class anti-deamidated gliadin-analog antibodies, GAF-3X) and endoscopy were performed to ensure CD diagnosis and to assess the intestinal mucosa status at the time of the diagnosis according to the Marsh scale, modified by Oberhuber [24]. These assessments were supplemented by a medical history of eating habits, based on a questionnaire about the frequency of usual intake of basic product groups during the week.

### 2.3. Assessments

All the adolescents were assessed for height and weight, and their body mass index (BMI) was calculated. Subsequently, BIA was performed with AKERN BIA-101 analyser (Akern SRL, Pontassieve, Florence, Italy) to examine their body composition and nutritional status. The measurements were performed between 7:00 a.m. and noon, on an empty stomach, in the supine position, with abducted upper (30°) and lower (45°) limbs, following at least a 5-min rest.

A tetrapolar electrode arrangement was applied with the contralateral recording mode. The amplitude of the measured current was 800 μA, sinusoidal, 50 kHz. To ensure the results were reliable and reproducible, two measurements were performed, one after another. Disposable electrodes were placed on the dorsal surface of the right arm (above the wrist) and the right leg (on the ankle). All measurements were performed according to guidelines described by other authors [25,26,27]. Dedicated software (Bodygram1_31 from AKERN, Pontassieve, Florence, Italy) was used to perform analyses of the results. The BIA took into account: fat mass (FM), fat free mass (FFM), muscle mass (MM) (kg and %), total body water (TBW), intra- and extra-cellular water (ICW and ECW) (litres and %), body cell mass (BCM) (kg and %) and body cell mass index (BCMI). Additionally, phase angle (PA) was calculated, based on resistance and reactance.

### 2.4. Statistical Analysis

Statistical analysis was performed using the Statistical Software for the Social Sciences SPSS Statistics 20 (IBM Software Group, San Francisco, CA, USA). For this purpose, parametric and non-parametric tests of significance were applied. Normality of the distributions of the quantitative variables was verified with the Kolmogorov–Smirnov test. Homogeneity of variances was then examined with the Levene’s test, and equivalence of variables distributions was verified using the χ^2^ test. If the conditions for application of parametric tests were fulfilled, it was possible to use the t-test for independent samples, one-way analysis of variance (ANOVA) or Pearson’s correlation. A *p*-value below 0.05 was considered statistically significant.

## 3. Results

Body composition parameters were significantly different between the CD and control groups. CD children had significantly higher mean values of the fat-free mass and total body water, and lower mean values of the fat mass (Table 2).

Among children with CD participants who were non-compliant to GFD presented apparently lower mean values of FM (kg) and also FM% and FFM%, than children who did follow dietary recommendations, but the difference did not reach the level of statistical significance (Table 3).

In a subset of 22 children and adolescents with CD, we performed a follow-up examination after a mean of 17.2 months. In this analysis children with CD demonstrated, as could be expected, significant weight, height and (borderline) BMI gain higher values of fat free mass, muscle mass, total body water (extracellular and intracellular), body cell mass and body cell mass index, while fat mass in kg did not increase significantly. None of these parameters expressed as a percentage of body composition has changed significantly during follow-up (Table 4).

Children and adolescents with CD who were non-compliant to GFD presented lower weight increase, and fall in BMI which was significantly different compared to group compliant to GFD. In addition, fat mass decreased, but the difference with participants who followed GFD did not achieve the level of statistical significance (Table 5).

## 4. Discussion

In the present case-controlled study, we observed that selected body composition parameters (fat mass, fat free mass, muscle mass, total, intracellular and extracellular body water, and body cell mass) and nutritional indicators (body mass index and body cell mass index) in children with CD are significantly different than in healthy controls. Reports related to changes in body composition, in adults and in children, both at the stage of diagnosis and during treatment, are limited and ambiguous. They depend on a number of variables, such as the age at the time of diagnosis, the disease progression, duration of impairments associated with malabsorption syndrome, methods of body composition assessment, as well as the degree of compliance to dietary guidelines.

The results of the present study showed that children with CD had significantly lower mean values of the FM expressed both in kg and as percentage of body mass (*p* = 0.007 and *p* = 0.015, respectively), and higher mean percentage values of the FFM (*p* = 0.015), and TBW (*p* = 0.012). Our results provide evidence that children with CD have lower energy reserves reflected as a lower total body fat mass, which may result in reduced immunity, a potential higher risk of malnutrition and faster dynamics of body components changes due to existing malnutrition. Furthermore, our results show, that children who were at baseline compliant to GFD presented apparently higher, but not statistically significant, mean values of FM and FFM (both in kg and in %), than children who did not follow dietary recommendations. The described differences in body composition components were close to the level of statistical significance despite the relatively low number of children in each group. After mean 17.2 months of follow-up, children with CD, as could be expected, demonstrated a significant increase of FFM, MM, TBW, BCM, and BCMI expressed in kg or L. However, the percentage of body mass components did not change significantly. Within this group children who did not follow strictly GFD presented lower weight gain and even decrease in BMI which was significantly different compared to the children compliant to GFD (*p* = 0.034 and *p* = 0.021, respectively). In addition, changes in fat mass tended to be different between compliant and non-compliant groups, which did not achieve statistical significance due to a very small number of children in each group. This indicates lower energy reserves and increased the potential risk of malnutrition in case of exacerbation of the disease in children not following GFD. Some studies report no significant changes in the specific components of body composition after a GFD is introduced [28], or, in fact, describe a decrease in fat-free components coinciding with stable fat mass one year after gluten withdrawal [19]. Other studies, including long-term research, provide evidence that after a GFD is introduced, the majority of the components of body composition are stable [17,18], sometimes with a slightly higher increase in FM than in FFM [29].

Our findings support suggestions made by other authors who agree that the earlier the diet is introduced, the faster it is possible to reverse abnormalities in body composition [18]. Important and constructive opinions regarding the necessity of the strict adherence to a GFD are voiced by studies which show that children, who fail to follow the recommendations, are found to have a significantly lower bone mineral density, which leads to a risk of osteoporosis in adulthood [30,31].

Another important, yet controversial issue, is the effect of a GFD in anthropometric parameters, in particular, the value of weight and BMI. Dyspepsia and malabsorption associated with progressing CD lead to malnutrition in quantitative and qualitative terms [32,33], which results, e.g., in both delayed growth and puberty [34]. Due to this, until recently, these patients were identified exclusively with low BMI. Currently, we know that over time CD may be accompanied by normal as well as excessive body mass, or even by obesity [35,36,37], because a GFD may contain both a high energy and high fat load [32]. The effects of a GFD, related to BMI vary greatly. Numerous studies show that the strict GFD results in normalization of BMI in initially underweight children and adults [28,35,38,39], and leads to significantly improved and faster growth and development in children, if the disease is diagnosed and treated early [40]. Conversely, in individuals with excessive body mass, at the time of CD diagnosis, BMI tends to decrease after the diet is introduced [39]. One should keep in mind that CD is a very heterogeneous disease, and the occurrence of the diagnosis among obese patients is no more surprising [37]. Our findings could be highly influenced by the duration of the disease and adherence to the GFD.

Based on current research it should be emphasized that adherence to a GFD is of critical importance in the treatment of CD and the further prevention of related complications. However, in order to achieve satisfying results, it is necessary to ensure the consistent monitoring of dietary restrictions, in combination with a systematic assessment of the patient’s nutritional status and body composition. Despite this, most patients with CD respond to a GFD, approximately 20% of them have persistent or recurrent symptoms. Following a GFD can be difficult for patients with CD and understanding the barriers/challenges experienced by patients in maintaining a GFD is essential for compliance [41]. There is also evidence suggesting that adherence to GFD is not enough, particularly in adolescent patients [42,43]. Our studies present 36.5% (15/41) non-compliant patients and our findings are similar to UK cross-sectional study in a group of patients with CD recruited from two London children’s hospitals. The high number of noncompliant participants 35.3% (36/102) indicates that maintaining GFD is difficult for practical and social reasons [44]. There are many studies evaluating not only the number of patients who do not follow the GFD but also analyzing the reasons for this noncompliance and their consequences for the quality of life [45]. Furthermore, a poorly balanced GFD may lead to deficiencies and, consequently, nutritional imbalance, which is particularly important in the case of children as it adversely affects growth, development, and physical activity. By monitoring the diet and by applying the simple methods to assess anthropometric parameters and indices, as well as body composition, it is possible to quickly identify and adequately correct any effects of nutritional errors, such as selective deficiencies of nutrients, as well as obesity, and to prevent health-related consequences.

Our study is obviously not free from several limitations. Despite our best efforts and inclusion in our study of as many participants as was possible, the relatively low number of study participants is the most important limitation. It did not allow us to find other significant differences between study and control groups as well as between participants with CD following and not-following GFD. Because assessment of important markers of nutritional status (albumin and/or pre-albumin level) was not done in all the children, we were not able to analyze the association of these variables with body composition components. Searching for such relationships would be an intriguing implication for further research. We also did not perform serology markers assessment at the follow-up visit. It would be interesting to analyze its relationship with compliance to GFD.

Given the controversies related to changes in anthropometric indices and body composition observed in individuals following a GFD, this problem unquestionably requires further study. It would be of extreme importance to conduct a long-term assessment of the effects produced by a GFD on body composition, beginning from childhood, when patients receive CD diagnosis until they reach adulthood. It would then be easier to eliminate small fluctuations in the components of body composition associated with age and the level of gluten cross-contamination in foods, and consequently to obtain reliable results.

## 5. Conclusions

The children with celiac disease differ significantly in body composition compared to healthy comparators. Participants adhering to a gluten-free diet presented a trend towards higher indices of selected body composition components. To assess the predictive and prognostic value of these findings, further prospective, longer-lasting studies, including a higher number of participants are required in this population.

## Figures and Tables

**Table 1:** Anthropometric parameters of the study and control groups. Significant differences in bold.

Parameter	Celiac Disease (*N* = 41)		Control (*N* = 41)		*p Value*
	Mean	SD	Mean	SD	
Age, years	10.81	3.96	10.63	4.01	0.989
Gender, n					1.000
Male	21	n/a	21	n/a	
Female	20	n/a	20	n/a	
Weight, kg	33.59	13.79	39.70	15.25	0.046 *
Height, cm	137.62	21.68	144.20	19.63	0.167
BMI, kg/m^2^	16.94	2.65	18.29	3.49	0.089

* indicate significant values (*p* < 0.05); BMI-body mass index; SD-standard deviation; n/a—not applicable.

**Table 2:** Results of BIA in the study and control groups.

Parameter	Celiac Disease (*N* = 41)		Control (*N* = 41)		*p Value*
	Mean	SD	Mean	SD	
FM, kg	6.66	4.19	9.47	5.15	0.007 *
FFM, kg	26.15	10.72	30.24	11.57	0.098
MM, kg	17.17	7.45	19.55	8.10	0.168
TBW, L	22.71	10.96	23.73	8.72	0.312
ECW, L	9.33	3.47	10.28	3.73	0.246
ICW, L	12.35	4.86	13.55	5.16	0.170
BCM, kg	13.89	6.11	15.85	6.64	0.164
FM%	19.32	7.36	23.34	7.36	0.015 *
FFM%	80.68	7.36	76.66	7.36	0.015 *
MM%	50.72	5.98	48.94	5.62	0.168
TBW%	65.22	8.94	60.47	7.66	0.012 *
ECW%	43.86	5.18	43.82	3.59	0.981
ICW%	56.14	5.18	56.18	3.59	0.981
BCM%	50.66	3.96	51.62	3.73	0.373
BCMI	6.91	1.28	7.22	1.50	0.322
PA	5.45	0.67	5.63	0.69	0.241

SD—standard deviation; FM—fat mass; FFM–fat free mass; MM—muscle mass; TBW–total body water; ECW—extracellular water; ICW—intracellular water; BCM—body cell mass; BCMI—body cell mass index; PA—phase angle; * Bold characters indicate significant values (*p* < 0.05).

**Table 3:** Anthropometric parameters and bioelectrical impedance (BIA) results among patients following and non-compliant to gluten free diet.

Parameter	Compliant to GFD (*N* = 26)		Non-Compliant to GFD (*N* = 15)		*p Value*
	Mean	SD	Mean	SD	
Age, years	11.00	4.10	10.47	3.82	0.683
Gender, n					0.239
Male	11	n/a	10	n/a	
Female	15	n/a	5	n/a	
Disease duration, months	74.23	58.10	26.07	40.93	0.002 *
Marsh scale					0.584
IIIA	4	n/a	3	n/a	
IIIB	9	n/a	7	n/a	
IIIC	13	n/a	5	n/a	
Weight, kg	35.67	14.07	29.99	12.96	0.208
Height, cm	141.02	21.73	131.73	21.00	0.190
BMI	17.22	2.55	16.45	2.84	0.272
FM	7.48	4.24	5.24	3.82	0.064
FFM	28.19	11.01	22.62	9.53	0.110
MM	17.95	7.63	15.83	7.17	0.388
TBW	22.49	8.00	23.08	15.12	0.675
ECW	9.65	3.56	8.76	3.35	0.434
ICW	12.91	4.67	11.37	5.18	0.457
BCM	14.52	6.26	12.79	5.89	0.390
FM%	20.81	6.60	16.73	8.10	0.087
FFM%	79.19	6.60	83.27	8.10	0.087
MM%	49.88	5.60	52.17	6.52	0.241
TBW%	63.89	8.69	67.52	9.19	0.214
ECW%	43.27	4.15	44.88	6.64	0.345
ICW%	56.73	4.15	55.12	6.64	0.345
BCM%	50.80	3.18	50.41	5.17	0.745
BCMI	6.91	1.16	6.90	1.50	0.978
PA	5.47	0.58	5.43	0.83	0.862

GFD—gluten-free diet; SD—standard deviation; FM—fat mass; FFM—fat free mass; MM—muscle mass; TBW—total body water; ECW—extracellular water; ICW—intracellular water; BCM—body cell mass; BCMI—body cell mass index; PA—phase angle; * Bold characters indicate significant values (*p* < 0.05); n/a—not applicable.

**Table 4:** Anthropometric parameters and BIA results among 22 patients (14 boys) followed for mean 17.2 months.

Parameter	Baseline (*N* = 22)		Follow-Up (*N* = 22)		*p Value*
	Mean	SD	Mean	SD	
Age, years	10.05	4.08	11.41	4.08	<0.001 *
Disease duration, months	63.68	67.61	80.86	68.14	<0.001 *
Weight, kg	32.40	15.68	36.01	14.08	<0.001 *
Height, cm	134.50	24.62	142.14	23.14	<0.001 *
BMI, kg/m^2^	16.81	2.76	17.07	2.09	0.046 *
FM, kg	7.20	4.62	7.42	3.75	0.101
FFM, kg	25.20	12.17	28.59	11.91	0.001 *
MM, kg	16.05	8.45	18.33	8.24	<0.001 *
TBW, L	20.16	9.25	22.81	9.02	<0.001 *
ECW, L	8.63	3.89	9.83	3.95	0.003 *
ICW, L	11.63	5.61	12.98	5.28	<0.001 *
BCM, kg	12.99	6.93	14.85	6.74	<0.001 *
FM%	22.05	6.50	21.17	6.94	0.972
FFM%	77.95	6.50	78.83	6.94	0.972
MM%	48.78	5.76	49.96	5.76	0.167
TBW%	62.66	8.50	63.39	9.00	0.455
ECW%	43.79	5.03	43.56	4.40	0.788
ICW%	56.21	5.03	56.44	4.40	0.788
BCM%	50.40	3.58	51.13	3.19	0.102
BCMI	6.60	1.26	6.88	1.12	0.005 *
PA	5.40	0.64	5.54	0.61	0.121

SD—standard deviation; FM—fat mass; FFM—fat free mass; MM—muscle mass; TBW—total body water; ECW—extracellular water; ICW—intracellular water; BCM—body cell mass; BCMI—body cell mass index; PA—phase angle; * Bold characters indicate significant values (*p* < 0.05).

**Table 5:** Differences in anthropometric parameters and body composition between 17 patients compliant and 5 patients non-compliant to gluten-free diet during follow-up.

Parameter	Compliant to GFD (*N* = 17)		Non-Compliant to GFD (*N* = 5)		*p Value*
	Mean	SD	Mean	SD	
Weight increase, kg	4.16	6.65	1.74	0.40	0.034 *
Height increase, cm	8.12	5.47	6.00	3.37	0.426
BMI increase, kg/m^2^	0.47	2.13	-0.44	0.78	0.021 *
FM increase, kg	0.47	3.66	-0.64	2.06	0.078
FFM increase, kg	3.69	4.90	2.38	2.39	0.308
MM increase, kg	2.51	3.26	1.48	1.86	0.182
TBW increase, L	2.45	3.21	3.34	3.47	0.597
ECW increase, L	1.37	2.04	0.64	0.60	0.209
ICW increase, L	0.96	1.45	2.70	2.92	0.610
BCM increase, kg	2.05	2.64	1.22	1.57	0.240
BCMI increase	0.31	0.72	0.20	0.53	0.289
PA increase	0.14	0.41	0.12	0.40	0.919

GFD—gluten-free diet; SD—standard deviation; FM—fat mass; FFM—fat free mass; MM—muscle mass; TBW—total body water; ECW—extracellular water; ICW—intracellular water; BCM—body cell mass; BCMI—body cell mass index; PA—phase angle; * Bold characters indicate significant values (*p* < 0.05).
